# Crystallographic Texture and Substructural Phenomena in 316 Stainless Steel Printed by Selective Laser Melting

**DOI:** 10.3390/ma16124289

**Published:** 2023-06-09

**Authors:** Ricardo Santamaria, Mobin Salasi, William D. A. Rickard, Kod Pojtanabuntoeng, Garry Leadbeater, Mariano Iannuzzi, Steven M. Reddy, Md Zakaria Quadir

**Affiliations:** 1 Curtin Corrosion Centre, Curtin University, Perth, WA 6102, Australia; ricardo.santamar@postgrad.curtin.edu.au (R.S.); mobin.salasi@curtin.edu.au (M.S.); thunyaluk.pojtanabuntoeng@curtin.edu.au (K.P.); g.leadbeater@exchange.curtin.edu.au (G.L.); mariano.iannuzzi@curtin.edu.au (M.I.); 2 Microscopy and Microanalysis Facility, John de Laeter Centre (JdLC), Perth, WA 6845, Australia; w.rickard@curtin.edu.au; 3 School of Civil and Mechanical Engineering, Curtin University, Bentley, WA 6102, Australia; 4 School of Earth and Planetary Sciences, Curtin University, Bentley, WA 6102, Australia; s.reddy@curtin.edu.au

**Keywords:** selective laser melting (SLM), 3D printing, additive manufacturing (AM), 316 stainless steel (SS316), EBSD, TEM

## Abstract

There is a fast-growing interest in the use of selective laser melting (SLM) for metal/alloy additive manufacturing. Our current knowledge of SLM-printed 316 stainless steel (SS316) is limited and sometimes appears sporadic, presumably due to the complex interdependent effects of a large number of process variables of the SLM processing. This is reflected in the discrepant findings in the crystallographic textures and microstructures in this investigation compared to those reported in the literature, which also vary among themselves. The as-printed material is macroscopically asymmetric in terms of both structure and crystallographic texture. The <101> and <111> crystallographic directions align parallel with the SLM scanning direction (SD) and build direction (BD), respectively. Likewise, some characteristic low-angle boundary features have been reported to be crystallographic, while this investigation unequivocally proves them to be non-crystallographic, since they always maintain an identical alignment with the SLM laser scanning direction, irrespective of the matrix material’s crystal orientation. There are also 500 ± 200 nm columnar or cellular features, depending on the cross-section, which are generally found all over the sample. These columnar or cellular features are formed with walls made of dense packing of dislocations entangled with Mn-, Si- and O-enriched amorphous inclusions. They remain stable after ASM solution treatments at a temperature of 1050 °C, and therefore, are capable of hindering boundary migration events of recrystallization and grain growth. Thus, the nanoscale structures can be retained at high temperatures. Large 2–4 μm inclusions form during the solution treatment, within which the chemical and phase distribution are heterogeneous.

## 1. Introduction

Selective laser melting (SLM) is a powder-based 3D printing/additive manufacturing (AM) technique for fabricating complex metallic parts with custom-designed internal and/or external structures. In SLM, a digital system drives a high-power laser beam, up to 1 kW, along a predesigned track to melt and fuse metallic powder particles layer by layer to build a complex metal/alloy shape and/or internal structures that otherwise would be impossible to fabricate with conventional metal processing techniques. SLM was initially developed almost two decades ago [1], but until recently, it has primarily been used in laboratory-scale and industrial prototyping [2,3,4,5]. Over the last ten years, the manufacturing sector has shown a keen interest in using SLM for industrial mass production. This is primarily due to improvements in SLM printing hardware, e.g., laser precision, powder manufacturing, etc., thus reducing time and production costs, as well as increasing the inherent metallurgical benefits of SLM manufacturing [2,3,4]. SLM provides a high degree of freedom in alloy compositions, covering both conventional and exotic mixtures, e.g., high-entropy alloys, and provides for post-heating treatments [6,7,8]. There are also unique metallurgical benefits in terms of the lightweighting and strengthening of material via the control of solidification rates and compositional gradients. These benefits are not possible, or are highly restricted, in conventional metal casting and subsequent thermomechanical processing (TMP) [9,10,11]. As a result, the SLM technique is rapidly being incorporated into industrial manufacturing [12,13,14], particularly in the aerospace, automotive, biomedical and energy sectors [15,16,17].

The metallurgical process variables between conventional metal processing and SLM are significantly different [18,19]. Consequently, the material properties, structures (at macro, micro and nano scales), and application performance have large differences, even for the same alloy composition. Nevertheless, it should be noted that the majority of metal AM techniques are developed from the concept of conventional techniques such as casting, welding, powder processing and/or cladding. These conventional techniques are not ideal when developing additive manufacturing process parameters and variables, and Aboulkhair et al. [18] recently summarized the SLM process variables, and their differences compared to conventional processing. For instance, castability and weldability are considered the primary characteristics for a given alloy’s suitability for SLM fabrication. Indeed, there are marked differences between the solidification rates and conventional welding parameters and those involved in SLM. Likewise, the remelting and rewelding during subsequent SLM scanning creates a thermal effect that has some similarities to conventional TMP [5].

Our current SLM knowledge is limited to a handful of alloy systems, including aluminum [19,20,21,22] and titanium [10,23,24] alloys, as well as some studies on stainless steel [25,26,27,28], nickel [29,30,31,32], cobalt [33,34,35,36], copper [37] and magnesium [38] alloys. Consistent and systematic investigations are essential to developing a detailed understanding of the effect of process variables on the microstructures and ultimate physical properties of SLM-printed materials; as such, it has taken many decades of research to reach the current level of knowledge for a given alloy system for a given conventional processing. The SLM journey has commenced, and the processes, microstructures and properties of materials processed in this way are in high demand because of the significant benefits and rapid growth of the technique. This paper presents a comprehensive analysis of the crystallography and composition of structures in an SLM-printed 316 stainless steel (SS316) in the macro to the nano scales for advancing and rectifying our understanding on the structural and crystallographic texture phenomena.

## 2. Materials and Methods

**Figure 1:**
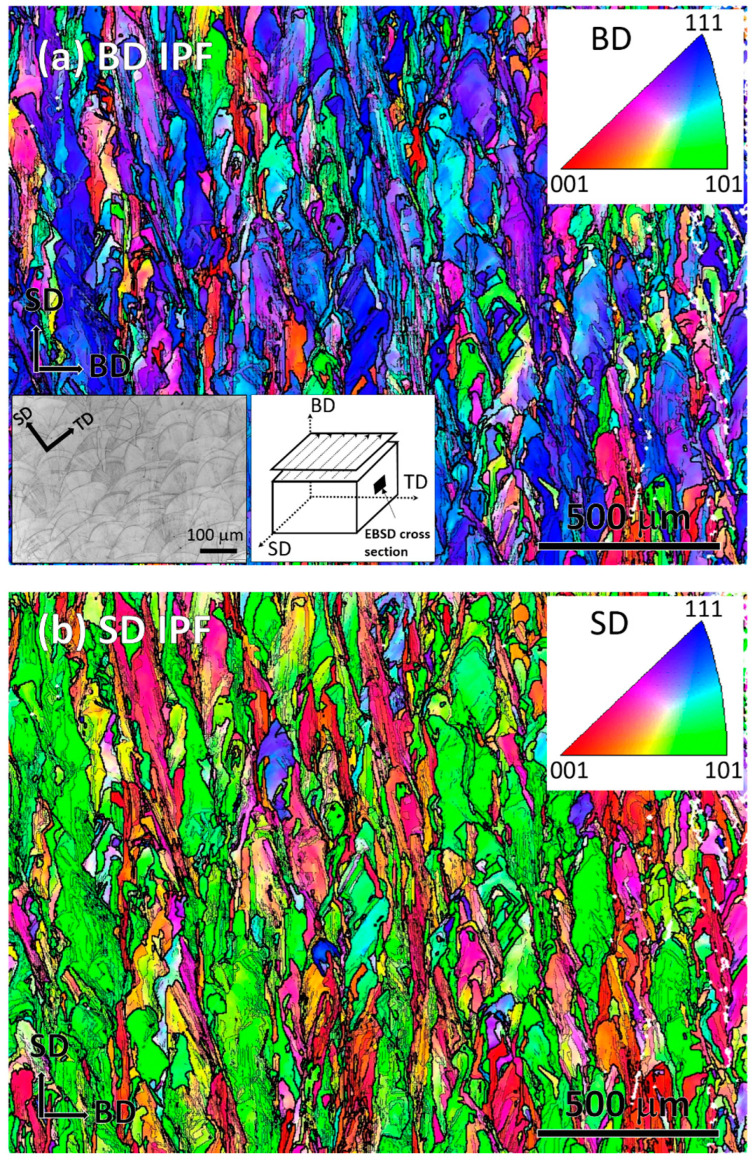
EBSD-measured color-coded IPF map of the as-printed SLM sample showing the orientation along the (**a**) BD and (**b**) SD. The insets in Figure 1a show the optical microscopy image and the EBSD cross-section.

SLM printing was conducted with a 3D system Pro X DMP 320 machine by a commercial 3D printing service company (Amiga Engineering, Tullamarine, VIC, Australia). A SS316 powder feed supplied by TLS Technik GmbH & Co (Bitterfeld-Wolfen, Germany) with 45 ± 10 µm average size was used to print a rectangular block 40 × 40 × 2 mm (X:Y:Z), see the schematic in Figure 1a inset. The other SLM parameters were 30 µm layer thickness, 250 W laser power, 900 mm/s laser speed, 30 µm scan resolution, parallel raster pattern with 0° rotation, and high-purity argon as the shielding gas. The feed chemical composition was provided by the supplier as 0.02 wt.% C, 0.51 wt.% Si, 1.0 wt.% Mn. 17.5 wt.% Cr, 2.3 wt.% Mo, 11.1 wt.% Ni, and Fe balance.

The printed material was subjected to an isothermal solution treatment at 1050 °C for 4 h in an argon-purged furnace followed by immediate water-cooling. The longitudinal cross-section, see the schematic in Figure 1a inset, of both the as-printed and heat-treated samples were cut, mechanically polished down to colloidal silica finish, and then ion milled with a Technoorg Linda SEMPrep II system (Budapest, Hungary) to obtain a defect-free surface suitable for investigation by electron backscattered diffraction (EBSD) investigation. The ion milling parameters were 8 kV, 6° tilt and 360° rotation. EBSD was conducted with an Oxford Instruments Symmetry^TM^ system attached to a Tescan Mira (Brno, The Czech Republic) field emission scanning electron microscope (FE SEM) operated at 15 kV beam energy. Iron FCC (face-centerd cubic) phase from the Oxford database was used for indexing EBSD patterns and the Oxford Instruments’ AztecCrystal software v.2.1.259 was used to post-process and analyze the EBSD data.

Site-specific transmission electron microscopy (TEM) samples were prepared from bulk samples using a Tescan Lyra Ga^+^ (Brno, The Czech Republic) focused ion beam (FIB)-SEM. The final polishing step was performed with a low beam energy of 2 kV to minimize ion beam damage. A FEI Talos FS200X G2 (Waltham, MA, USA) FE TEM was used for the TEM investigation and was operated at 200 kV. Elemental mapping was conducted by an energy dispersive spectroscopy (EDS) attached as two pairs of a FEI Super X detection system. Location-specific diffraction analysis was performed using selective area diffraction (SAD) with an aperture with a diameter of 200 nm and convergent beam electron diffraction (CBED). A double tilt holder was used to tilt the sample to the intended crystallographic zone axis by navigating through the CBED generated Kikuchi pattern. TEM imaging was conducted both in conventional and scanning TEM (STEM) modes. For STEM, the bright field (BF) and high-angle annular dark-field (HAADF) mode were used to enhance diffraction and atomic number contrast, respectively. TEM data acquisition and analysis was undertaken using Velox software and diffraction data analysis was conducted using the international center for diffraction data (ICDD) database.

## 3. Results and Discussion

### 3.1. Structural Symmetry and Crystallographic Texture

Figure 1a,b show EBSD color-coded inverse pole figure (IPF) maps in the building direction (BD) and scanning direction (SD) in a BD-SD cross-section from an SLM-printed block. The investigated cross-section schematic is shown in the inset in Figure 1a, in which the terminology of the orthogonal print axes is shown, in convention with comparable studies, e.g., [39]. In Figure 1, the optical micrograph shows the characteristic print features in an SD-TD surface previously reported in numerous investigations [39,40,41,42]. From the IPF maps, it is clear that the BD and SD were predominantly oriented along the <111> and <101> crystal directions, respectively. It has been well-established that crystallographic texture in iron controls its anisotropy in mechanical, thermal, magnetic and optical properties. The observed macroscopic crystallographic texture is therefore likely to play a fundamental control on the anisotropy of physical properties in SLM-printed SS316. The thick and thin black lines in the EBSD map represent the high-angle (>15°) and low-angle (3–15°) misorientation boundaries, respectively. The high-angle boundaries are broadly parallel to the laser scanning tracks associated with printing. There was no evidence of the formation of the ∑9 twin boundary (<111>60°) in the as-printed sample. This finding is consistent with the other literature, where no twin boundaries were reported in SLM-printed SS316, although the wrought form of the material contained annealing twins [41,42].The formation of twin boundaries is generally promoted by low solidification rates [43,44]. Therefore, in the case of SLM-manufactured SS316, where solidification occurs almost instantly at cooling rates ranging from 10^3^ to 10^7^ K/s [2,45,46,47], the formation of twin boundaries is hindered. This rapid cooling prevents sufficient time for the atoms to rearrange, which in turn delays the nucleation and growth of twin boundaries. This is in contrast to wrought SS316L, where cooling rates are much slower and facilitate the formation of twin boundaries [41,42].

In the IPF map presented in Figure 1b, there are thin <001>-oriented layers, colored in red, between thicker <101>-oriented printing tracks, colored in green. These green and red layers are called ‘major’ and ‘minor layers’ by Sun et al. [48] (pp. 89–93). The thickness of the major and minor layers varied between 100 and 200 μm and 50 and 100 μm, respectively, suggesting an overall crystallographic relationship between the major and minor layers. In both IPF maps, there are other orientations in the major layers, which are present in the red and green regions in Figure 1a, and red and blue regions in Figure 1b. EBSD analysis in the TD revealed mixed orientation, not presented in the Figure 1. These findings suggest that a sample-scale macroscopic crystallographic texture forms in SLM-printed SS316, which is consistent overall with the recent literature, but the reported textures vary in terms of crystal orientation [48,49,50,51]. An epitaxial growth mechanism between the major and minor layers is regarded as the origin of the overall texture development [48]. However, lattice epitaxy requires a perfect match between two lattice interfaces with common coincident sites, which is somewhat unrealistic to imagine in the SLM-printed material, because it contains a continuous change in orientation, as reflected by a gradual change in color within short distances in the EBSD maps. Hence, a separate in-depth investigation at a finer length scale is required to find out whether there are epitaxies over short distances, and if this collectively develops the overall texture.

**Figure 2:**
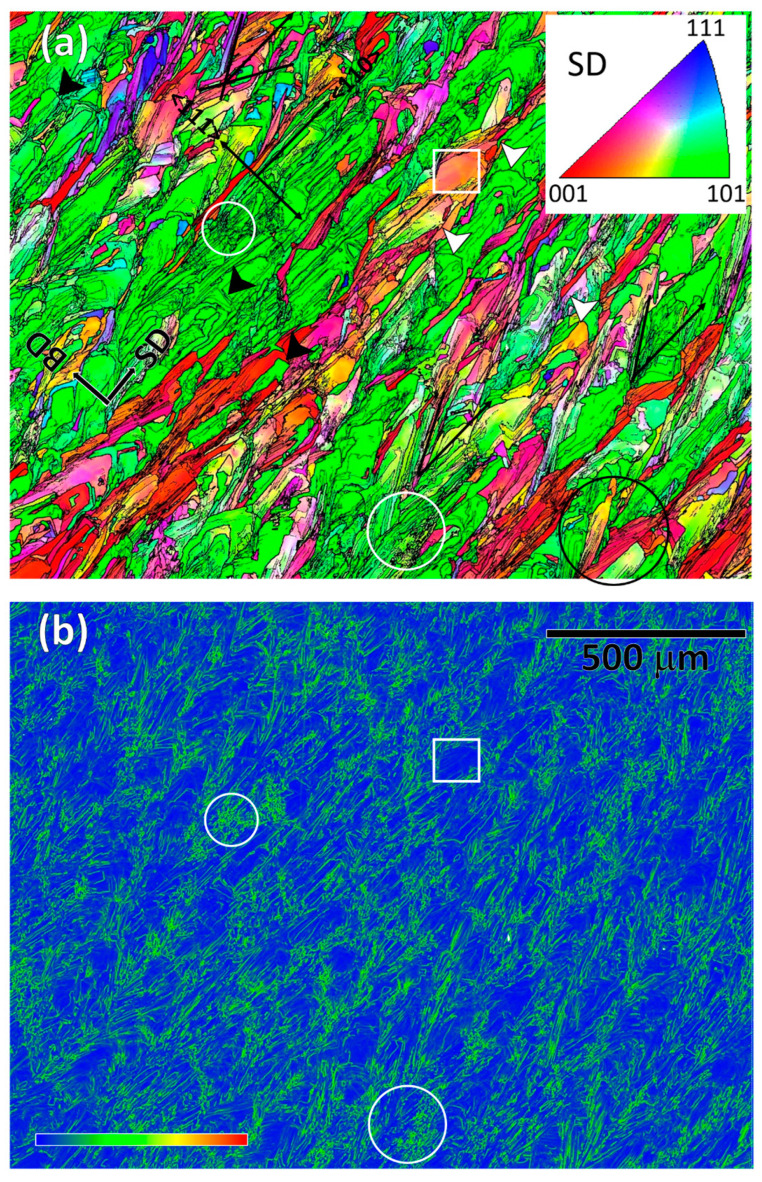
Higher-resolution EBSD maps of the as-printed SLM sample showing (**a**) color-coded IPF map of SD to illustrate the inhomogeneities in the high (>15°) and low-angle (3–15°) boundary distributions and their alignments with the SD and (**b**) the corresponding inhomogeneities in the KAM plot.

In Figure 2a, the misorientation boundaries are elucidated in a higher-resolution, 100 nm step size, IPF map, whereby the SD is plotted as per the color-coded IPF section in the inset. As before, the high- (>15°) and low-angle (3–15°) boundaries are represented by thick and thin black lines, respectively. The corresponding Kernel average misorientation (KAM) map is shown in Figure 2b, in which each data point represents the mean orientation difference with the eight surrounding neighboring points. The blue–yellow–red legend in Figure 2b indicates the relative KAM intensity. There is a correlation between Figure 2a,b, viz., comparison of the white encircled areas shows that the high-stored-energy spots have a higher density of misorientation boundaries. This observation can be understood in relation to dislocation density because a higher dislocation density is required to accommodate any misorientation. There were also regions of low stored energy. One such example is encompassed with a white rectangular box, within which there is a small orientation variation, represented by a minor change in the IPF color variation. Such low misorientation variations indicate the presence of dislocation mesh and cell structures, which usually accommodate relatively less energy [52]. Therefore, the as-printed sample showed an overall heterogeneous distribution of the stored energy. This finding explains the spatial variation of the micro- and nano-scale mechanical data in the SLM-printed material [53,54].

Figure 2a also reveals several other morphological features of the boundary. For example, the majority of the boundaries were straight, though there were several high-angle boundaries that had convoluted trajectories, some of which are indicated with white arrows. This phenomenon indicates the occurrence of a thermally induced restoration process, perhaps from the heat flow from the subsequent SLM scanning [52]. It is important to note that the boundaries depicted in Figure 2 are also present in Figure 1. However, Figure 1 provides a broader field of view, making the details of the boundaries less apparent. Figure 2 complements Figure 1 by offering a closer view (or finer scale) that provides additional information about the boundary features. There was no sign of recrystallization, as noted by an absence of a trailed region with a uniform orientation behind a migrating high-angle boundary [52]. The convoluted high-angle boundaries are expected to have formed during solidification or due to subsequent thermal restoration [52], although the process did not progress to the boundary migration stage of recrystallization.

### 3.2. Substructural Features

There is a profuse presence of straight misorientation boundaries in Figure 2a, which are aligned, within a certain angular range, with the SD, as indicated by the black lines. Some straight boundaries are aligned along the SD, as shown in Figure 2a, which is <101> of the lattice direction. A small fraction also aligns at the right angle, in short segments indicated with the black arrows, which is along the BD ||<111>. The remainder, accounting for the largest fraction of straight boundaries, are aligned in the ±30–45° angular range, with the highest frequency being around ±35°. Some boundary combinations also resemble a leaf vein structure, with changing directions; one such example is circled in black at the right bottom of Figure 2a. Therefore, the overall alignment of the straight boundaries is rather complex, which Dinda et al. [55] (pp. 2152–2160) described as a function of the laser scanning strategy. In some recent studies, the boundaries appeared to have a coincidence with the crystallographic planes, most commonly along the {100} plane trace, e.g., SS316-, Ni-25% (Mo, Nb and Ti)-, Al-, Ta-, Ti-Mo-Zr-Al- and Mo-Si-alloys [48,49,55,56,57,58,59,60]. A few mechanisms for the formation of these textures have been outlined in the published literature based on the formation of the solid/liquid interface in order to explain their crystallographic origin. The scan rate and laser energy have been reported to play a vital role in this regard [61]. In this investigation, however, the alignment of the straight boundaries invariably remained identical within an angular range with the SD, irrespective of the matrix orientation, as shown in Figure 2a. For instance, the boundary orientations in the blue-, located in the upper left, and red-oriented regions comprise the same angular alignments with SD as the boundaries found in the vast majority green regions. This suggests that the low-angle straight boundaries are non-crystallographic, viz., they do not preferentially form on a particular lattice plane trace(s). Although this conclusion is made based on unequivocal evidence, it should be noted that only a 3D EBSD can reveal the real crystallography of a 3D interface. There is evidence that 2D trace analysis of 3D boundary features may lead to misleading conclusions. One such example is the low-angle microband boundaries that form in high stacking fault energy materials that have been claimed to be both crystallographic [62] and non-crystallographic [63]. This debate continued until reconciliation was achieved on the basis of a 3D EBSD investigation [64,65].

A recent article by Pham et al. [51] (p. 749) accounts for the variations in boundary formation in SLM-printed SS316, such as those seen in Figure 2a. The fundamental basis remains identical to the previous reports, viz., the boundaries form along the solid–liquid interface during the solidification process [48,66,67]. In Pham et al.’s simulation work, it was demonstrated that side branching occurs, similarly to the current findings shown in Figure 1 and Figure 2, during the solidification process, and thus alters the shape of the solidifying boundary front. As a result, the alignment of the solidification interface changes, and therefore, the formation of low-angle boundaries takes place over a wider angular range. The magnitude of side branching depends on a number of factors, primarily on the thermal gradient and heat flux, and the SLM parameters that control these two. Each narrative in the literature on low-angle crystallographic boundary formation, including Pham et al.’s study, is overwhelmed by the assumption that the solid/liquid interface appears as a crystallographic lattice interface. However, physical details on the mechanism by which the habit plane or rotation axis correlate with a preferred crystal plane or direction are missing. Therefore, the mechanism of low-angle boundary formation is rather complicated, because of the simultaneous occurrence of rapid solidification with the complex mechanical interaction of the semi-solid pool by laser beam movement. In addition, there is thermal pulsing during the subsequent overlay of layers.

**Figure 3:**
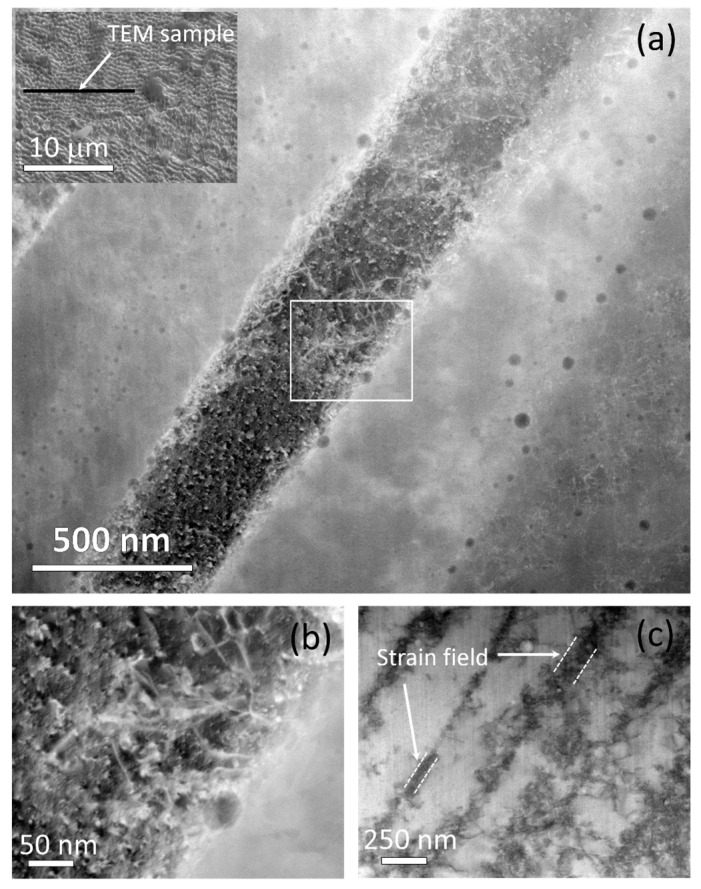
HAADF STEM images showing (**a**) particle decorations in the dislocation-constituted boundaries of the commonly found fine cell structures (in the inset) in the SLM-printed sample. (**b**) Particle pinning at the dislocation boundary in a higher-magnification HAADF STEM image of the white rectangular area located in subfigure (**a**)**.** (**c**) BF STEM image showing the strain field width of the dislocation boundaries at the boundaries edge on titled condition.

Numerous investigations have reported columnar structures that also appear as fine cellular structures in the transverse cross-section of SLM-printed SS316 [40,68,69]. An example of such a cell structure is shown as an SEM image in the inset of Figure 3a. Unlike the low- and high-angle boundary structures in Figure 1 and Figure 2, this structure was found homogeneously throughout the sample. These cellular structures have been reported to vary in size from 0.25 to 1.2 μm, with the actual size having an inverse relationship with the laser scanning speed. [46,70,71]. It has been observed that these structures exhibit a weaker strengthening effect compared to the grain boundaries of the microstructure [71]. Because of the submicron-scale fineness of the cellular structures, an electron-transparent TEM sample was prepared using FIB-SEM site-specific lift-out methods. Figure 3a shows an HAADF STEM image of the TEM sample, in which the columnar structures are sub-vertical in the cross-sectional lamellae. The walls of the columns are densely populated with dislocations, and the walls are spaced parallel at an average distance of 500 nm. These boundaries were also decorated with 5–30 nm spherical particles. The particles were tangled within the boundary dislocations, see higher magnification image in Figure 3b, and created a pinning effect. These particles are likely to have restricted any thermally activated migration, and thus, restricted the structures to the nanoscale. The dislocation walls were 50–150 nm in thickness and are expected to have created a strain field, which became apparent through the diffraction contrast in the BF STEM imaging in Figure 3c, which was taken after tilting the sample so that the boundaries were edge-on. These dislocation features are expected to provide elevated strengthening in the SLM-printed SS316 material over the conventionally processed grade that usually comprises large equiaxed grains, hundreds of µm in size, and twin boundaries. This is reflected in a 20–50% improvement in the tensile strength of SLM-printed SS316 over the conventional grade with an identical chemical composition [40]. The strength can also be improved by changing the laser strategy that works at a larger length scale. While further discussions of mechanical properties are outside the scope of this paper, it is expected from the results presented herein that superior strengthening at the micro and nano scale can be achieved in SLM-printed grade due to the retention of nanostructures and the formation of inclusions due to the rapid cooling (~10^3^–10^5^ K/s) of SLM solidification [72].

**Figure 4:**
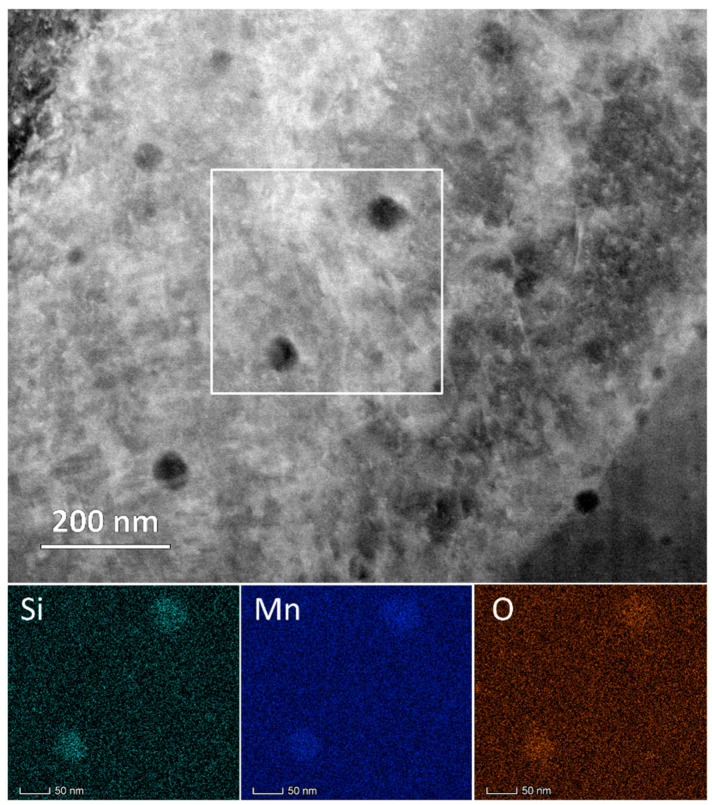
HAADF STEM image (**top**) of the nanoparticles and corresponding EDS-measured elemental maps (**bottom**) of the white rectangular area, showing that the particles are rich in Si, Mn and O content in the as-printed SLM sample.

The darker appearance of the particles in the HAADF STEM images in Figure 3 indicates that they had a lower average atomic weight than the matrix. In Figure 4, an area was selected that contained larger particles, and these were subjected to elemental analysis by STEM-EDS. Elemental maps revealed that the particles were rich in Mn, Si and O. Significant efforts were devoted to determining the crystallographic identity of the inclusions using SAD and CBED diffraction techniques, but no diffraction spots were observed other than those from the FCC iron matrix, and therefore, these particles are likely amorphous. This finding is consistent with the report by Salman et al. [69] (pp. 205–212). It is pertinent to note that Shibata et al. [73] (pp. 522–528) found larger particles, ~1 µm, with identical morphologies in cast SS316. These were characterized as MnO–SiO_2_ particles, solely based on the chemical ratio measured by electron probe microanalysis and thermodynamic calculations. In some cases, they also found a small association of Cr_2_O_3_. In regard to the current study, it is important to note that Cr was not measured within the particles, and no Cr-C crystalline diffraction patterns were observed. Therefore, Cr is expected to remain in the solid solution to provide the intended stainless property in the SLM-printed SS316.

### 3.3. Solution Treatment Structures

A solution treatment at 1050 °C for 4 h, per ASM [74] recommendations, of the as-printed sample is expected to anneal any thermally unstable microstructures, and to ensure a uniform Cr dissolution into the matrix. It is pertinent to note that the stainless properties are impaired in conventional-grade SS316 because of the inadequate presence of atomic Cr in the solution that occurs due to Cr-C formation. The solution treatment brings the Cr atoms back to the matrix as solutes. Cr-containing inclusions were not observed in the samples in this study, see Figure 3 and Figure 4, which suggests that the solution treatment is not needed for Cr dissolution purposes in the SLM material. However, the heterogeneous boundary structures shown in Figure 2 may result in an uneven Cr distribution, because dislocations are naturally preferable sites for solute atoms. Therefore, the solution treatment may indeed promote an even Cr concentration.

**Figure 5:**
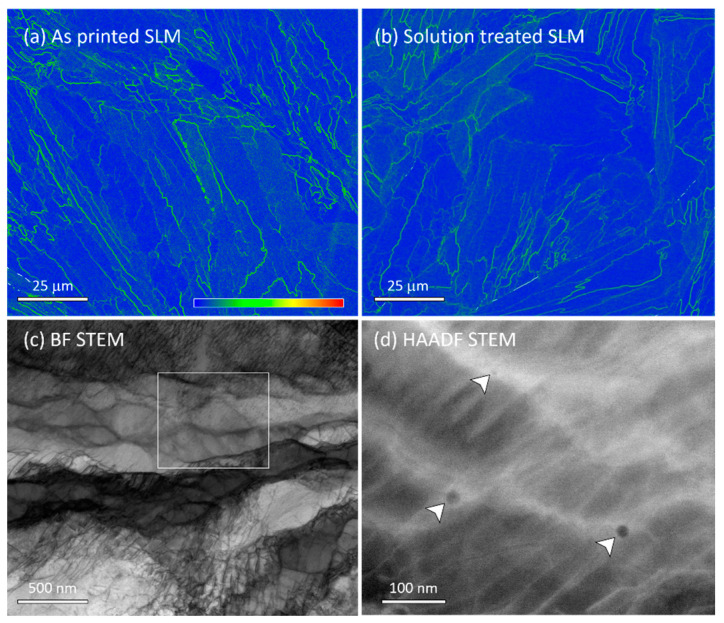
EBSD-measured high-resolution KAM map showing the differences in the stored energy distributions between the (**a**) as-printed and (**b**) solution-treated SLM samples in the BD-SD cross-section. TEM investigation of the solution-treated sample shows (**c**) the changes in the dislocation boundary structures in a BF STEM image and (**d**) the retention of boundaries by particle (arrowed) pinning in a magnified HAADF STEM image of the rectangular area marked in subfigure (**c**).

Interestingly, only a subtle change took place in the substructures during the 1050 °C solution treatment. Figure 5a,b present a comparative view in the form of KAM maps that reveal an overall reduction in the KAM-intensive boundary density after the solution treatment. The solution-treated structure is also shown in the BF STEM micrograph in Figure 5c, in which the dislocation-constituted boundaries underwent a thermal relaxation process, compared with Figure 3, viz., the boundaries were curved and the dislocations were dissociated. The rectangular area in Figure 5c is magnified in the HAADF STEM image in Figure 5d. Analysis revealed boundary pinning by the inclusions that were found in the as-printed sample in Figure 3 and Figure 4. They were measured to contain Mn, Si and O as per the as-printed sample. Overall, the inclusion density was significantly reduced by the solution treatment, perhaps because of some degree of dissolution and/or agglomeration. The high stability of the inclusions after the solution treatment at 1050 °C explains why recrystallization and grain growth did not take place in the SLM-printed material. Previously, Shibata et al. [73] (pp. 522–528) reported that amorphous Mn-Si-O particles remain stable even after 1200 °C solution treatment in cast SS316, where grain growth was not prevented because the density was low and the inclusions size was large, >1 μm.

**Figure 6:**
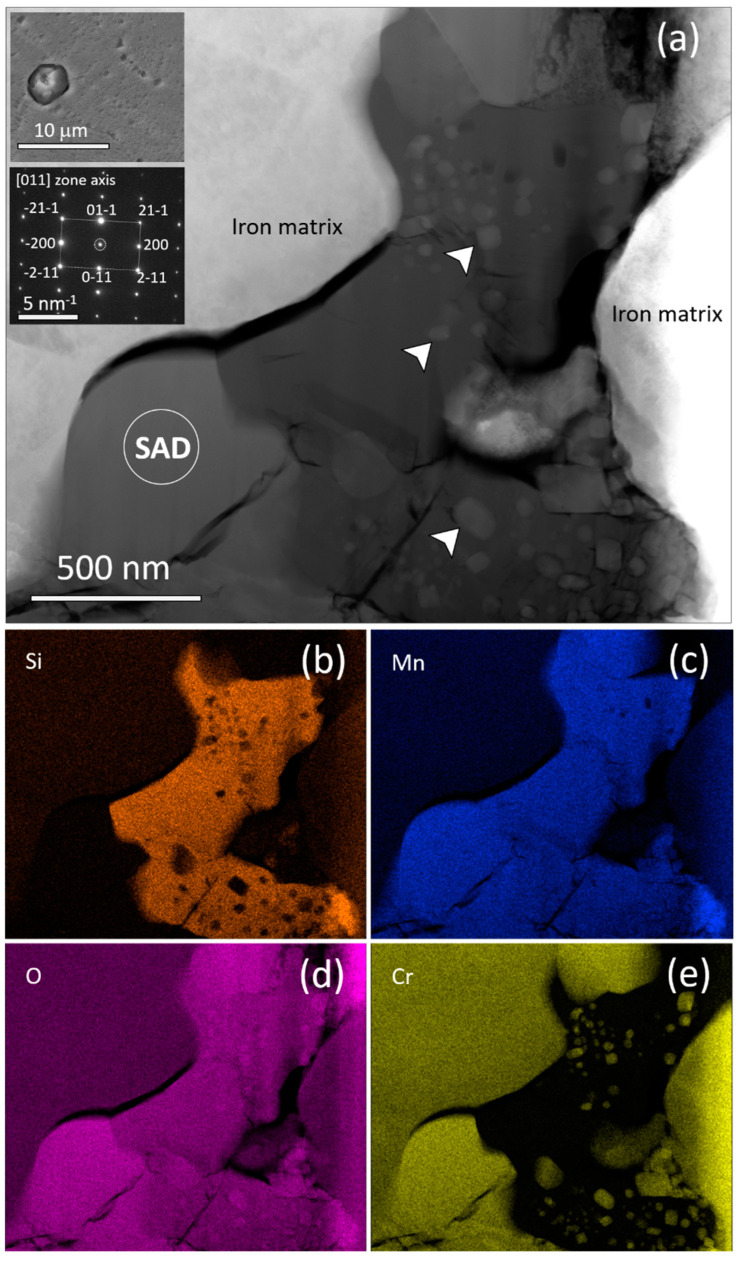
The structural and chemical heterogeneity of inclusions in the solution-treated SLM-printed sample is shown in (**a**) the HAADF STEM image, and (**b**–**e**) the corresponding elemental mapping for Si, Mn, O, and Cr, respectively. The insets in (**a**) show the inclusion from which the TEM sample was prepared, and the indexed SAD pattern of Cr_3_O_4_ from the SAD-labeled area.

It is important to note that inclusions 2–4 μm in size were also observed in the solution-treated sample that were absent under as-printed conditions. An example is shown in the upper inset in Figure 6a, whereby a TEM lamella was prepared by FIB and presented as a STEM HAADF image (Figure 6a) in order to determine the chemical distribution within the inclusion. The surrounding iron matrix appears brighter. It should be noted that it was identified via TEM-EDS that the inclusions were rich in Mn, Si, and O, although there were also Cr- and O-rich regions within the inclusions, which can also be seen as brighter regions, as indicated by arrows, in the darker overall matrix. An SAD pattern was taken of the marked area and indexed as Cr_3_O_4_, as illustrated in the lower inset of Figure 6a. The iron matrix also contained Cr, which was expected as the solute. These findings suggest that during the solution treatment, a large fraction of the nano-sized inclusions agglomerate into large 2–4 µm inclusions. The Cr from the solid solution also diffused and participated in the formation of inclusions, since Cr was not found in the inclusions in the as-printed sample. Overall, the localized corrosion resistance of the as-printed SLM-manufactured SS316 is excellent, which can be attributed to factors such as the absence of sulphides, the presence of a more stable passive film, lower rates of metastable pitting, and a higher pitting potential compared to its wrought counterpart [75,76,77]. However, the presence of these large inclusions with heterogeneous chemical and structural distribution after heat treatment, as reported in this work, has been shown to be detrimental to the alloy’s resistance to localized corrosion by decreasing its pitting potential [78]. Therefore, the solution treatment recommended by ASM [74] for conventional SS316 is, indeed, expected to be detrimental to the SLM-printed material.

SLM-manufactured SS316 components have shown excellent mechanical properties [40,79,80] and localized corrosion resistance [75,76,77], often surpassing their conventionally manufactured wrought counterparts. Therefore, it is crucial to evaluate their performance against common damage mechanisms observed in the energy sector, such as stress corrosion cracking (SCC) or hydrogen-induced cracking (HIC), in both as-printed and heat-treated microstructures. Additionally, exploring the optimization of properties through variations in printing parameters or the utilization of post-processing steps would be of great significance.

## 4. Conclusions

In this investigation, a thorough microscopic characterization of SLM SS316s/s under as-printed and solution-annealed conditions was conducted at the macro, micro and nano scales. The findings suggest some of the existing findings are inconclusive or imprecise, and require further investigation to mature our knowledge in this area. The conclusions of this study can be summarized as follows:

1. SLM-printed material possesses an asymmetric crystallographic texture and material structure. The microstructure has a distinctive structural morphology along the orthogonal axes of the sample, and develops crystallographic textures in SD ||<101> and BD ||<111>.
2. Heterogenous distributions of misorientation boundaries and stored energy were found throughout the SLM-printed structures. Twin boundary formation was not observed in either the as-printed or solution-annealed samples.
3. In the as-printed structures, the typical straight misorientation boundaries were characterized as being non-crystallographic. The boundaries maintained general alignment with the SD within an angular range, irrespective of the matrix’s crystal orientation, although there were occasional coincidences with crystal plane traces.
4. The high-angle boundaries in the SLM substructures underwent thermal restoration, which was activated by the heat originating from the printing of the subsequent layer. Pinning by the nano inclusions hindered classical recrystallization, and thus, prevented the formation of a defect-free annealing structure, even after 4 h of solution treatment at 1050 °C.
5. A nano-scale lamellar structure with a width of 500 ± 200 nm formed homogeneously throughout the printed material. Depending on the orientation, the structures appeared with cellular or columnar morphologies in SEM and TEM images. Their boundaries contained dense dislocation structures tangled with fine amorphous inclusions containing Mn, Si and O. Cr was not found above the limit of detection in the inclusions. Hence, Cr remains in the matrix to provide the stainless properties.
6. Some degree of dissociation of the dislocation boundaries occurred during the solution treatment, but the overall refined structures were retained. Additionally, inclusions with a size of 2–4 μm formed, consisting of composite structures and chemical distributions. These inclusions can have detrimental effects on the localized corrosion resistance of the alloy.

## Data Availability

This article contains all the data that was generated and is presented in the form of Figures.
